# Small Vessel Ischemic Disease of the Brain and Brain Metastases in Lung Cancer Patients

**DOI:** 10.1371/journal.pone.0007242

**Published:** 2009-09-30

**Authors:** Peter J. Mazzone, Nicola Marchi, Vince Fazio, J. Michael Taylor, Thomas Masaryk, Luke Bury, Tarek Mekhail, Damir Janigro

**Affiliations:** 1 Respiratory Institute, The Cleveland Clinic, Cleveland, Ohio, United States of America; 2 Neurological Institute, The Cleveland Clinic, Cleveland, Ohio, United States of America; 3 Florida Hospital Cancer Institute, Orlando, Florida, United States of America; 4 Cell Biology, The Cleveland Clinic, Cleveland, Ohio, United States of America; National Cancer Institute, United States of America

## Abstract

**Background:**

Brain metastases occur commonly in patients with lung cancer. Small vessel ischemic disease is frequently found when imaging the brain to detect metastases. We aimed to determine if the presence of small vessel ischemic disease (SVID) of the brain is protective against the development of brain metastases in lung cancer patients.

**Methodology/Principal Findings:**

A retrospective cohort of 523 patients with biopsy confirmed lung cancer who had received magnetic resonance imaging of the brain as part of their standard initial staging evaluation was reviewed. Information collected included demographics, comorbidities, details of the lung cancer, and the presence of SVID of the brain. A portion of the cohort had the degree of SVID graded. The primary outcome measure was the portion of study subjects with and without SVID of the brain who had evidence of brain metastases at the time of initial staging of their lung cancer.109 patients (20.8%) had evidence of brain metastases at presentation and 345 (66.0%) had evidence of SVID. 13.9% of those with SVID and 34.3% of those without SVID presented with brain metastases (p<0.0001). In a model including age, diabetes mellitus, hypertension, hyperlipidemia, and tobacco use, SVID of the brain was found to be the only protective factor against the development of brain metastases, with an OR of 0.31 (0.20, 0.48; p<0.001). The grade of SVID was higher in those without brain metastases.

**Conclusions/Significance:**

These findings suggest that vascular changes in the brain are protective against the development of brain metastases in lung cancer patients.

## Introduction

Brain metastases occur in approximately 15% of all cancer patients [1]–[3]. Ten –15% of patients with lung cancer have brain metastases at diagnosis, and an additional 20%–25% develop brain metastases during their illness [4]. Guidelines suggest brain imaging at presentation in asymptomatic lung cancer patients with evidence of locally advanced non-small cell carcinoma, all patients with small cell carcinoma, and anyone with symptoms that could be related to the presence of brain metastases (e.g. headache, seizures) [5]. Computed tomography or magnetic resonance imaging (MRI) of the brain is performed in these situations.

Metastatic spread to the brain is a multi-step process. To produce brain metastases, tumor cells must: 1. reach the brain vasculature, 2. attach to the endothelial cells, 3. extravasate into the parenchyma, 4. proliferate, 5. induce angiogenesis, and 6. avoid immune surveillance [6]. Thus one might surmise that the survival and proliferation of metastases to the brain relies on a healthy and recruitable blood supply. It is generally assumed that the mechanisms underlying CNS immunoprivilege, the blood-brain barrier, acts also as a natural barrier to metastases. Intuitively, one may then predict that a leaky BBB will favor metastatic recruitment of systemic tumors to the CNS. A leaky BBB also removes the immunoprivilege. CNS immunoprivilege may *favor* metastatic growth; in fact, a recent report has shown that activation of brain immunity may decrease or delay metastatic growth [7]. In addition, recent findings have shown that brain-specific processes allow extravasation of tumor cells across the intact BBB, thus revealing a unique mechanism that promotes extravasation under condition of intact vasculature [8]. Finally, a recent manuscript has shown that a leaky BBB does not necessarily allow better CNS access for small molecules such as antiepileptic drugs [9]. Thus, while the BBB is a formidable shield protecting the brain, its failure does not necessarily lead to complete loss of function.

Over the past decade, imaging of the brain has improved. We are now able to identify subclinical vascular changes in the brain, termed small vessel ischemic disease (SVID). Narrowing of the vascular lumen and failure of cerebral autoregulation result in ischemic damage of the cerebral white and subcortical gray matter [10]. Lacunar brain infarcts and cerebral white matter lesions are examples of findings related to SVID [11]–[17]. These lesions are commonly observed on MRI scans of elderly people and are associated with an increased risk of stroke, dementia, and depression [18]. Clinical factors known to increase the risk of SVID include increased age, hypertension, diabetes mellitus (DM), hyperlipidemia, and cigarette smoking. Some of these are shared risk factors for the development of lung cancer.

In a previous study, we found that a large proportion of patients diagnosed with lung cancer were also found to have SVID on staging brain MRI [19]. Given the central role of the vasculature in the development of brain metastases, we aimed to determine if the presence of SVID was protective against the development of metastases to the brain of individuals with lung cancer.

## Results

**Figure 1:**
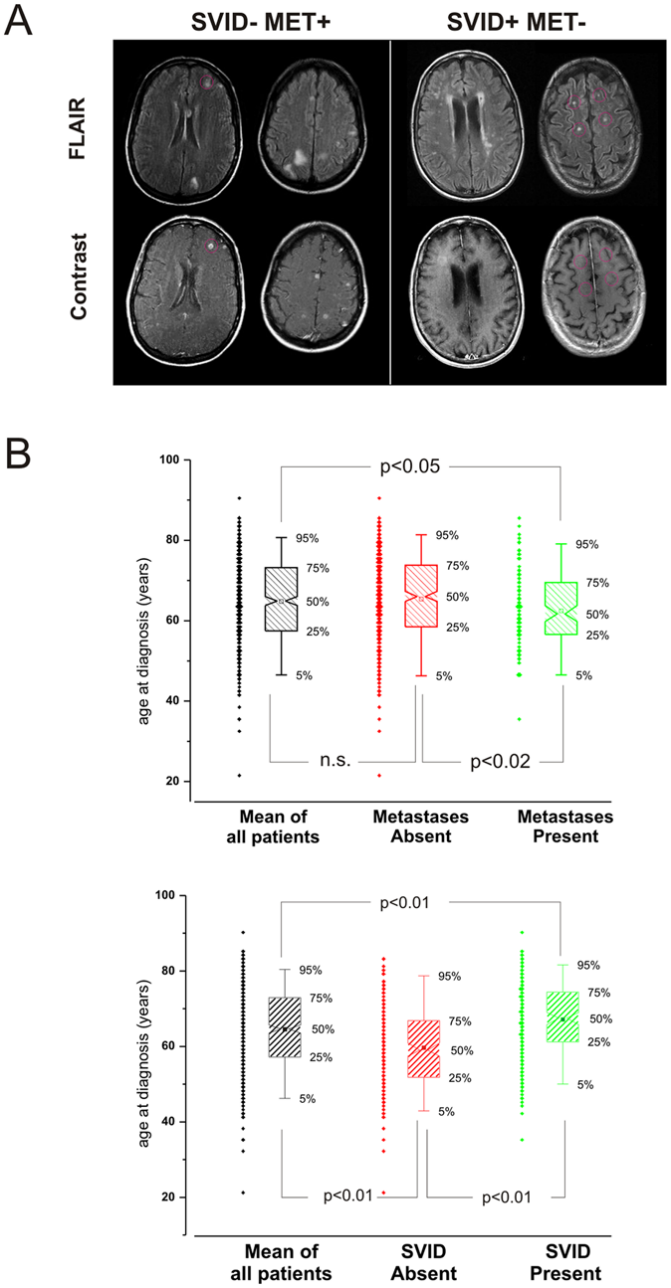
Comparison of SVID and metastases by MRI and age: A) Radiologic evaluation of SVID and metastases was based on comparison of post-contrast and FLAIR images. Note that metastases were obviously demarcated after gadolinium (Gd) injections, while SVID visible in FLAIR were not. The *red circles* refer to the locations of SVID or metastases in FLAIR or post-Gd images. B) Age distribution of patients affected by SVID or metastases. Patients with no metastases were younger than those with metastatic brain tumor; patients with SVID were significantly older than those without small vessel disease.

A total of 523 patients were enrolled in the study. Data from phase 1 of the study, including the patients' demographics and co-morbidities are summarized in conjunction with the presence and absence of brain metastases and SVID in Table 1. The criteria for detection of metastasis is summarized in the Methods section and exemplified in Figure 1A. The presence of brain metastases was evaluated after contrast injection. Typically, brain metastases presented as highly enhancing lesions with variable degree of perilesional edema. The topographic relationship between metastatic brain tumors and white matter hyperintensities was also studied to emphasize that neoplastic lesions rarely occurred in proximity of primary brain edema presumably pre-existent to the metastatic invasion.

**Table 1:** Characteristics of lung cancer patients compared between those who presented with and those who presented without brain metastases.

Study Characteristic	All	Brain Metastases	No Brain Metastases	P-Value
**Number**	523	109	414	–
**Age (mean years,±SD)**	64.6+/−10.5	62.1+/−9.6	65.3+/−10.7	0.001
**Male Sex (%)**	54.5	57.8	53.6	0.44
**Tobacco Use (%)**	90.6	89.9	90.8	0.77
**Diabetes Mellitus (%)**	11.5	7.3	12.6	0.13
**Hypertension (%)**	40.9	37.6	41.8	0.43
**Hyperlipidemia (%)**	28.5	21.1	30.4	0.055
**SVID (%)**	66.0	44.0	71.7	<0.0001

SD = standard deviation, SVID = small vessel ischemic disease.

At the time of lung cancer presentation 109 patients (20.8%) had evidence of brain metastases on MRI, and 345 (66.0%) were reported to have evidence of SVID. Individuals without brain metastases were older (65.3+/−10.7 years vs. 62.1+/−9.6 years, p = 0.001). There was a trend towards a lower likelihood of brain metastases in patients with hyperlipidemia (present in 30.4% of those without metastases vs. 21.1% of those with metastases, p = 0.055) or DM (present in 12.6% of those without metastases vs. 7.3% of those with metastases, p = 0.13).

At the time of lung cancer presentation, individuals with SVID were older (67.2±9.4 years vs. 59.6±10.7 years, p<0.001), more likely to have a tobacco use history (92.8% in those with SVID vs. 86.5% in those without SVID, p = 0.02), and more likely to have hypertension (47.0% in those with SVID vs. 29.2% in those without SVID, p<0.0001). There was a trend towards increased SVID if hyperlipidemia was present (31.0% in those with SVID vs. 23.6% in those without SVID, p = 0.07).

**Figure 2:**
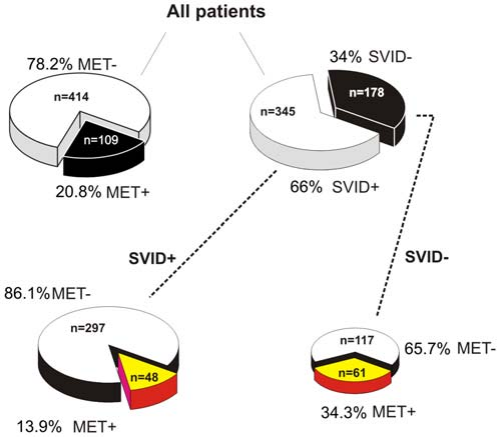
Pie chart comparison of metastatic and SVID patient groupings: This figure provides a summary of results on incidence of metastases in patients affected or not by small vessel ischemic disease. See text for details.

Study subjects with brain metastases at presentation were less likely to have SVID (44.0%) than were those without brain metastases (71.7%) (p<0.0001, Figure 2). Put another way, 13.9% of those with SVID and 34.3% of those without SVID presented with brain metastases (p<0.0001). In a multivariate model that included age and co-morbidities, the presence of SVID was found to be the only protective factor against the development of brain metastases, with an OR of 0.31 (0.20, 0.48; p<0.001). Many brain metastases (defined as 5 or more) were seen in 27.5% of those with brain metastases, 34.4% of those without SVID vs. 18.8% of those with SVID (p = 0.07).

**Figure 3:**
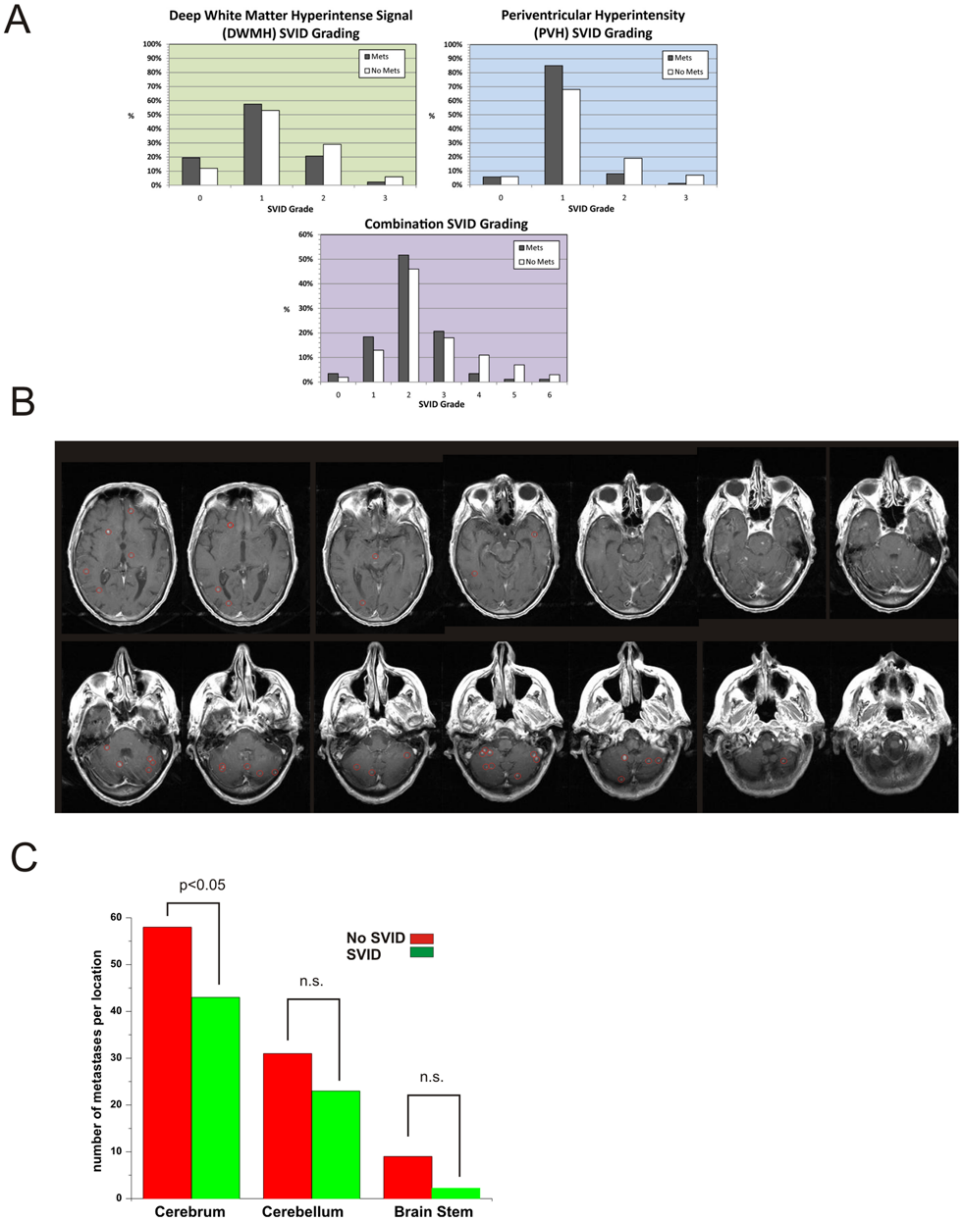
SVID grading methods, brain metastases identification, and metastatic distribution: A) Grading for SVID: deep white matter hyperintense signals, periventricular hyperintensity, and combined. Each represents the SVID distribution of grades of those with and without brain metastases. Differences were significant for deep white matter hyperintensity (p = 0.04), periventricular hyperintensity (p = 0.01), and the combined (p = 0.02). B) MRI image with gadolinium contrast demonstrates the protocol used to count identifiable metastases. These are indicated by empty red circles. C) Distribution of metastases in different CNS regions. Note that in the region where SVID are most common (cerebrum) there was a statistically significant difference in the number of metastases as predicted by a protective effect of SVID against tumor growth. See text for details.

In phase 2 of the study, the degree of SVID was graded for selected study subjects as described in the methods. Two common radiologic manifestation of SVID is the presence of deep white matter hyperintensities, periventricular hyperintense signals or both. Figure 3A shows that those without brain metastases who were graded for SVID had more SVID (83.0% vs. 68.2%, p = 0.004), and were more likely to have hyperlipidemia (42.0% vs. 26.8%, p = 0.005), and be older (69.2±10.0 years vs. 64.1±10.6 years, p<0.001) than those without brain metastases who were not graded. Those with brain metastases who were graded for SVID showed a trend towards a greater frequency of SVID (48.3% vs. 27.3%, p = 0.08). They were otherwise statistically similar.

The frequency of SVID grades for deep white matter hyperintense signals (DWMH), periventricular hyperintense signals (PVH), and the combination of these two are shown in Figure 3 for those with and without metastases in whom they were graded. Those without brain metastases had higher DWMH grades of SVID (p = 0.04), higher PVH grades of SVID (p = 0.01), and higher combination grades of SVID (p = 0.02) than those with brain metastases.

**Figure 4:**
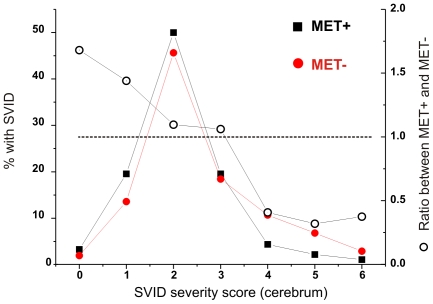
Relationship between SVID severity and metastatic brain tumor: The data are presented as % (filled symbols) or as a ratio between SVID severity in the two subsets of patients.

We then focused to the anatomical location of metastases in SVID positive or negative patients (Figure 3B and Figure 4). Patients with SVID had, on average, fewer metastases as expected if a negative correlation exists between SVID and metastatic tumor. This was true for all the regions where metastases were measured. in the region where SVID are most common (cerebrum) there was a statistically significant difference in the number of metastases as predicted by a protective effect of SVID against tumor growth. We then measured the severity of SVID in relation to the presence of metastases. As shown in Figure 4, patients with metastatic tumors had a much lower SVID score.

## Discussion

The main finding of our study is that lung cancer patients with SVID in the brain have a lower likelihood of having brain metastases than those without SVID. This was demonstrated by the following findings: 1) Patients with SVID had a lower likelihood of presenting with a brain metastasis; 2) SVID was protective against brain metastases even after controlling for SVID risk factors (hypertension, age, DM, tobacco use, and hyperlipidemia); 3) The higher the grade of SVID, the more protection there was against presenting with a brain metastasis; and 4) There was a trend towards those with brain metastases having fewer metastases if SVID was present. To our knowledge, this is the first report to describe this relationship.

The reason that brain metastases are less common in those with SVID is not fully understood. The “soil and seed” theory of metastases states that a cancer cell must leave its original tumor location and establish in a hospitable environment for a metastasis to develop [20]. Thus, a cirrhotic liver would be an unlikely site for metastases to develop since the blood supply is poor and metabolic byproducts and inflammatory infiltrates are present that could interfere with tumor cell growth [21]. There are several aspects of brain histology and physiology that make it an ideal site for metastatic growth, while other factors are present that would prevent metastases from developing. Facilitating factors include the absence of strong innate and acquired immunity in the brain parenchyma, the absence of lymphatic drainage, and the abundant supply of oxygen and glucose. In contrast, the ability of the blood-brain barrier and the tight junctions of endothelial cells to isolate the brain from the systemic circulation serves to protect the brain from developing metastases [22]. One potential explanation for the protective role of SVID is that the local immunity in an area of vascular change may be altered. Altered local immunity could occur if the blood-brain barrier function was impaired in an area of metastasis-induced neo-vascularization, allowing enhanced leukocyte, antibody, and complement infiltration into the area, providing acquired immunity to an organ that does not normally have it [23]. A second potential explanation of the protective effect of SVID is that a change in the vascular architecture may lead to an inability of the cancer cell to extravasate, receive nutrition, or induce angiogenesis. Changes in the vascular architecture, such as the changes that occur in the vascular basement membranes in long-standing DM, may impede the spread of tumor cells by making the basement membranes less digestible by tumor cell related proteinases. It has recently been suggested that DM can protect against metastases in those with lung cancer [24]. Others have debated a potential survival advantage in individuals with DM who develop malignancies [25], [26]. Our results point to the vascular changes, rather than the underlying condition that leads to the vascular changes, as the reason for protection against brain metastases.

There are potential problems with our study. The retrospective design influences the accuracy of the data. Though DM, hypertension, and hyperlipidemia have well known definitions their listing in the electronic medical record may not have been entirely accurate or complete. Grading of SVID suggested a higher prevalence of SVID than was noted on the MRI reports. The MRI reports may have been less likely to describe SVID when an obvious metastasis was present or when the SVID was only very mild. Those with co-morbidities and SVID may be more likely to receive routine medical care, leading to earlier identification of their cancer, with a lower likelihood of having a brain metastasis at presentation. We believe that the strength of the evidence from multiple lines of reasoning (prevalence of metastases, number, and relation to grade of SVID) support our conclusions despite these concerns. Finally, our study only assessed patients with lung cancer. We cannot conclude that our findings would apply to other malignancies known to metastasize to the brain.

A possible confounding aspect of population studies on human disease is comorbidity. In our population we were able to associate the presence of SVID to several other factors (diabetes, etc.). This may lead to a different life span in the subjects, and therefore add additional variability. We only included brain metastases which were present and diagnosed at the time of the lung cancer diagnosis (at initial staging). Since we did not include brain metastases that developed throughout the course of the patient's lung cancer, the fact that those with more comorbidities die earlier would not influence our results

In conclusion, our findings suggest that vascular changes in the brain are protective against the development of brain metastases in lung cancer patients. The use of staging tests and the choice of treatment relies on the application of clinical and molecular predictors of risk. Knowledge of the influence of a patient's vascular status on risk could be one more factor to consider in the management of a patient with lung cancer. Determining the nature of the observed protection will advance our understanding of lung cancer pathogenesis and provide insights into novel management strategies.

## Materials and Methods

### Study Design and Patients

The study was performed with the approval of the Institutional Review Board (IRB) at the Cleveland Clinic (IRB# 07-698). Written consent was provided by the patients enrolled in phase 1 of this study. The data obtained from phase 2 of this study was a retrospective analysis in which the IRB approved a waiver requiring written patient consent. In the first phase, the medical records of patients with lung cancer were reviewed. These patients were identified from two sources. The first group of patients had enrolled in a prior study that evaluated the diagnostic potential of serum markers of blood-brain barrier dysfunction in the diagnosis of cerebral metastases (91 patients) [19]. The second group of patients was sequential patients seen by a medical oncologist (Dr. Masaryk; 432 patients) from 6/05-6/07. All patients from both groups had biopsy proven lung cancer and had undergone MRI imaging of their brain as part of standard staging. Patients were not included if they had a history of another cancer diagnosed within 5 years of their lung cancer presentation (except for non-melanoma skin cancers and localized prostate cancer). Data collected included patient demographics, details of the lung cancer, and risk factors for the development of SVID (DM, hypertension (HTN), hyperlipidemia, and tobacco use). The presence of these risk factors was based on a clinical diagnosis listed in the electronic medical record. MRI reports from the initial evaluation of the lung cancer were reviewed for the presence of brain metastases and descriptions of SVID.

In the second phase, MRI scans were re-read under the guidance of a staff neuroradiologist (TM) with a focus on grading the severity of SVID. Eighty seven of those with metastases to the brain had MRI scans available for grading (the other 22 were outside studies). For those without metastases to the brain, 100 were chosen for grading. These 100 had the lowest medical record numbers of the group without metastases who had MRI scans available for review. The grading system used was a combination of previously described rating scales [27]–[30]. Periventricular hyperintensity (PVH) was graded as 0 = absence, 1 =  “caps” or pencil-thin lining, 2 = smooth “halo,” 3 = irregular PVH extending into the deep white matter. Separate deep white matter hyperintense signals (DWMH) were rated as 0 = absence, 1 = punctate foci, 2 = beginning confluence of foci, 3 = large confluent areas. These scores were analyzed separately and combined for each patient to give a total burden of SVID score.

### Statistical Considerations

Continuous measures were described as means, standard deviations and percentiles. Categorical measures were summarized using frequencies and percentages. Wilcoxon Rank Sum tests were used for the comparison of ordinal measures for binary outcomes. For the evaluation of association between categorical measures, Pearson's Chi-square test of Fisher's Exact test were used. The relationships between ordinal measures were evaluated by Spearman correlation coefficient and Pearson's correlation coefficient was used to assess the association between continuous measures. Logistic regression was performed to assess the association between ‘SVID grades’ and ‘mets’, after adjusting for confounding factors. All tests were performed at a significance level of 0.05. SAS 9.1.3 software (SAS Institute, Cary, NC) was used for all analyses. Additional statistical analysis was performed by ANOVA and t-test analysis to determine the influence of age on propensity toward brain metastasis or SVID.
